# Role of the Gastrocnemius Musculocutaneous with a Propeller Style Skin Flap in Knee Region Reconstruction: Indications and Pitfalls

**DOI:** 10.1055/s-0043-1768644

**Published:** 2023-08-24

**Authors:** Gianluca Sapino, Rik Osinga, Michele Maruccia, Martino Guiotto, Martin Clauss, Olivier Borens, David Guillier, Pietro Giovanni di Summa

**Affiliations:** 1 Department of Plastic and Hand Surgery, Centre Hospitalier Universitaire Vaudois (CHUV), Lausanne, Switzerland; 2 Department of Plastic, Reconstructive, Aesthetic and Hand Surgery, University Hospital of Basel, Basel, Switzerland; 3 Department of Plastic and Reconstructive Surgery, University Hospital of Bari, Bari, Italy; 4 Department of Orthopedic and Trauma Surgery, University Hospital of Basel, Basel, Switzerland; 5 Department of Orthopedic Surgery, Centre Hospitalier Universitaire Vaudois (CHUV), Lausanne, Switzerland; 6 Department of Plastic Reconstructive and Hand Surgery, Department of Oral and Maxillofacial Surgery, University Hospital, Dijon, France

**Keywords:** musculocutaneous gastrocnemius flap, medial sural artery perforator gastrocnemius flap, GM-MSAP, knee reconstruction, upper leg soft tissue reconstruction

## Abstract

**Background** Soft tissue reconstruction around the knee area is still an open question, particularly in persistent infections and multiple reoperations scenario. Flap coverage should guarantee joint mobility and protection, even when foreign materials are implanted. The chimeric harvesting of the musculocutaneous gastrocnemius flap, based on the sural artery perforators, can extend its applicability in soft tissue reconstruction of the upper leg, overcoming the drawbacks of the alternative pedicled flaps.

**Methods** A multicenter retrospective study was conducted enrolling patients who underwent to a pedicled, chimeric gastrocnemius musculocutaneous–medial sural artery perforator (GM-MSAP) or lateral sural artery perforator (GM-LSAP) flap for knee coverage in total knee arthroplasty (TKA) recurrent infections and oncological or traumatic defects of the upper leg from 2018 to 2021. Outcomes evaluated were the successful soft tissue reconstruction and flap complications. Surgical timing, reconstruction planning, technique, and rehabilitation protocols were discussed.

**Results** Twenty-one patients were included in the study. Nineteen GM-MSAPs and 2 GM-LSAPs were performed (soft tissue reconstruction in infected TKA [12], in infected hardware [4], and in oncological patients [5]). Donor site was closed primarily in 9 cases, whereas a skin graft was required in 12. Flap wound dehiscence (1), distal flap necrosis (1), distal necrosis of the skin paddle (1), and donor site infection (1) were the encountered complications. Flap reraise associated to implant exchange or extensive debridement was successful without requiring any further flap surgery.

**Conclusion** The propeller–perforator GM-MSAP offers qualitative defect coverage and easiness of multiple flap reraise due to skin availability and its laxity.

## Introduction

The coverage of the knee region represents a challenge for the plastic surgeon. Soft tissue defects can expose critical structures such as joint, bone, or tendon. The situation becomes even more complex when foreign material is present with dramatic consequences in terms of postoperative infection and implant contamination. 1

The reconstruction should aim to preserve the joint function and mobility, while being solid and prevent exposure recurrence. Various surgical techniques have been described so far to address this complex scenario, including local flaps, pedicled flaps, and free flaps. 2 3

The gastrocnemius muscle (GM) is considered the workhorse flap to reconstruct soft tissue defects in the knee area, with proven efficacy in both posttraumatic injuries and implant salvage. 4 5 The solid vascularity of the flap can guarantee an efficient antibiotic deliverance at the injured site while the ease of harvest and the reliable surgical anatomy do not require microsurgical expertise and is the base of its extensive use. 6

However, some limitations should be acknowledged: potential hypotrophy of the muscle component, limited vascularization at its distal region with difficulty to reach defects superolateral to the patella, necessity of skin grafting over the transposed muscle with postoperative immobilization to facilitate healing.

Last decades advancement in surgical anatomy knowledge has permitted to describe a wide number of fasciocutaneous flaps option based on perforators vessels. The musculocutaneous gastrocnemius flap, described in the early 70s by McCraw et al, has been recently rediscovered for knee reconstruction, with a new knowledge on skin perforator harvest possibilities (deriving from the sural artery system). 7 8 Such enhanced tailoring possibilities can expand flap potential for reconstruction, fully exploiting both muscle and skin components. 9 10

This study investigates knee reconstructions using chimeric gastrocnemius musculocutaneous–medial sural artery perforator (GM-MSAP) flaps in complex knee reconstruction where simple GM flap seemed insufficient, including recurring arthroplasty infections and extended sarcoma or traumatic defects. Outcomes and complications have been critically analyzed, together with surgical technique and planning.

## Methods

Prospectively maintained databases of patients treated at three University Hospitals from 2018 until 2021 were retrospectively searched for adult patients with knee or upper leg defects and soft tissue reconstruction (STR). Besides oncological cases (e.g., sarcoma resections), the majority of patients requiring STR of the knee were patients with infected total knee joint arthroplasties (TKA) or fracture-related infections. 11 12

Only patients who underwent STR with a pedicled, chimeric GM-MSAP or lateral sural artery perforator (GM-LSAP) flap were included in this study. The patient's demographic data and comorbidities as defined by the Charlson Comorbidity Index were recorded.

Informed consent was obtained from all patients, including approval for photographic\video documentation. Ethical institutional approval was granted: CER-VD 2022-00434

### Surgical Timing

In the oncological cases, the resection was followed by negative pressure dressing, with STR performed after confirmation of histopathological clean margins (generally after 7–10 d).

In infected TKAs or fracture-related infections, the duration of infection and the causing microorganism dictated the treatment concept (one- vs. two-stage procedure). Despite the stages, in patients of all three institutions STR was generally performed as early as possible. In patients who underwent a one-stage procedure the STR was performed directly after the debridement, antibiotics, and implant retention or after the implant exchange procedure. For patients who underwent a two-stage procedure, flap STR was performed directly after implant removal and spacer implantation during the first of the two stages. The rationale for this relied on the following arguments: first, early surgery maximizes the time for the soft tissue to heal and integrate. Furthermore, a well-vascularized reconstructed tissue can act as a vehicle for the transport of antimicrobial agents to the site of infection. However, this was not always possible as patients were often referred after first stage treatment.

### Outcome Analysis

The primary outcome was the successful STR. The first outpatient follow-up investigation was performed 3 weeks after STR. This was followed by 6 weeks, 3 months, and 6 months controls. Decisions on intravenous antibiotic treatment discontinuation were always multidisciplinary including the orthopaedics and the infectious medicine team. Flap reraise for second stage procedures were generally performed 6 to 8 weeks after debridement and cement spacer placement. In patients who underwent a two-stage procedure, further follow-ups were scheduled after reraising and reinsetting the flap during second stage. Successful STR was defined by the presence of an intact and dry soft tissue envelope.

The secondary outcome was related to flap complications. Complications were listed as major and minor, according to previous literature. 13 Major complications were considered full or partial flap necrosis (at least ⅓ of the flap, necessitating new flap procedures), whereas minor complications were considered partial flap necrosis (less than ⅓ of the flap, and maintained vascularization allowing STSG). Early complications were defined as complications occurring within 6 weeks after flap surgery (first or second stage). Late complications were defined as complications arising between 6 weeks and up to 3 years.

### Surgical Technique

Acoustic Doppler ultrasound was used as a starting point for preoperatively localizing reasonable perforators overlying the head of the GM. A line was drawn from the midpoint of the popliteal crease along the medial leg to the apex of the medial malleolus. A major perforator was usually found within a semicircle with a 2 to 3 cm radius, centered distal to a point along this line 8 cm from the popliteal crease. A second, more significant perforator was normally found more distally, within a circle with a radius of 2 to 3 cm centered on this same line 15 cm from the popliteal crease. The axis of the flap was oriented in a longitudinal direction paralleling the long bones of the leg, to capture adjacent perforasomes. 14

**Fig. 1:**
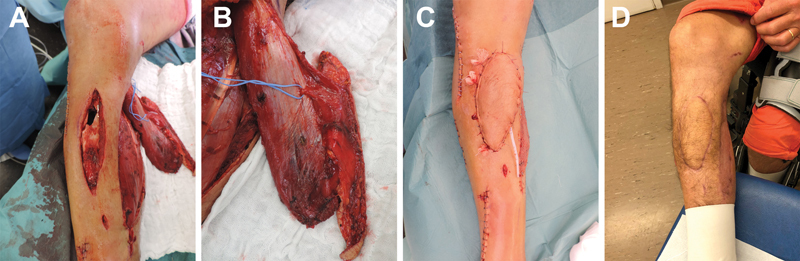
Patient no. 4 sustained a high energy trauma with Gustillo IIIA tibial fracture. The late infection osteosynthesis material required an extensive soft tissue debridement ( **A** ). a chimeric gastrocnemius MSAP was used for the soft tissue coverage ( **B** ). The soft tissue reconstruction at the end of the surgery ( **C** ) and at 6 months follow-up ( **D** ).

Loupe magnification was used for flap harvest. The incision started at midcalf, 2 cm behind the posterior border of the tibia on the medial aspect of the leg, and curved proximally in direction of the popliteal fossa. After opening the deep fascia, a subfascial dissection allowed fast visualization of perforators to the skin paddle, which were tested in terms of size and pulsatility. Intramuscular dissection of the chosen perforator was performed if needed to reduce the risk of perforator kinking or torsion after flap transposition ( Fig. 1 ). The skin incision around the skin paddle was then completed and the skin paddle was secured to the underlying muscle to avoid shear forces on the perforators during the muscle harvest.

Once the flap was harvested and rotated into the defect. In our experience, once the gastrocnemius turned medially the skin paddle will be further turned between 40 and 90 degrees, depending on the size and location of the defect.

**Fig. 2:**
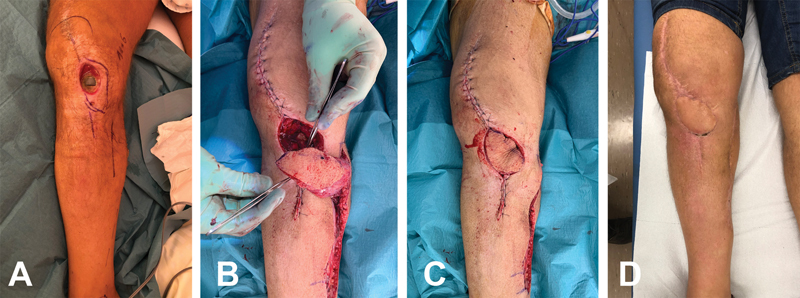
Patient no. 9 sustained an infection of a total knee prosthesis (TKA) ( **A** ). A two-stage surgical procedure was performed including extensive soft tissue debridement, TKA exchange with cemented spacer and soft tissue reconstruction using a chimeric gastrocnemius-MSAP ( **B** ), the skin flap was rotated to the defect with an angle of 45 degrees ( **C** ). Follow-up at 2 months ( **D** ).

Donor site was closed directly or skin grafted when the skin paddle was exceeding 7 cm in width ( Fig. 2 ).

### Postoperative Rehabilitation and Physiotherapy Protocols

Postoperatively, patients followed a protocol of 3 days in bed without cast immobilization. Orthostatic position was progressively achieved from day 3 to 7, associated with class II compression garments, and progressive knee joint mobilization (0–45 degrees from day 3–7). In patients with framework and nonconsolidated fractures, weight-bearing was progressively introduced according to orthopaedics, whereas patients with TKA were weight-bearing at 2 weeks with full flexion mobilization according to pain and under physiotherapy supervision.

## Results

During the study period, 21 patients (9 females) were included. Of these, 12 patients needed soft tissue reconstruction over an infected TKA, 4 over infected hardware of the lower leg, and 5 after sarcoma resection. Demographics and comorbidities are outlined in Table 1 .

**Table 1:** Patients data and characteristics

Pt. no.	Age/sex	BMI	Smoke	Diabetes	ASA score	Ethiology	Localization	Flap	Defect size (cm ^2^ )	Skin paddle size (cm ^2^ )	OP time (min)	FU (m)
1	72/M	25	N	Y	3	Infected TKA	Knee	GM-MSAP	80	100	135	30
2	64/M	28	N	N	3	Infection OM	Tibia	GM-MSAP	50	74	141	26
3	49/F	23	Y	Y	2	Infected TKA	Knee	GM-MSAP	45	60	200	25
4	63/M	20	N	N	1	Infection OM	Tibia	GM-MSAP	60	120	120	20
5	48/F	23	N	N	1	Infection OM	Tibia	GM-MSAP	48	70	180	19
6	62/M	29	N	Y	3	Infected TKA	Knee	GM-MSAP	35	70	330	7
7	55/M	27	Y	N	2	Infected TKA	Knee	GM-MSAP	48	60	150	7
8	42/M	27	Y	N	2	Sarcoma resection	Knee	GM-MSAP	200	180	190	26
9	88/F	25	N	N	2	Infected TKA	Tibia	GM-LSAP	15	50	160	15
10	85/F	35	N	N	3	Infected TKA	Tibia	GM-MSAP	36	50	145	20
11	80/M	31	N	N	2	Infected TKA	Tibia	GM-MSAP	100	129	210	25
12	76/F	28	N	N	3	Infected TKA	Tibia	GM-MSAP	70	90	240	18
13	25/F	19	N	N	1	Sarcoma resection	Knee	GM-LSAP	50	80	120	22
14	76/M	21	N	N	3	Infected TKA	Knee	GM-MSAP	15	20	211	12
15	50/F	31	N	N	3	Infected TKA	Knee	GM-MSAP	30	60	200	27
16	61/M	31	N	N	3	Infected TKA	Knee	GM-MSAP	54	60	279	30
17	27/F	34	N	N	3	Infection OM	Tibia	GM-MSAP	140	100	235	15
18	78/F	31	N	N	3	Infected TKA	Knee	GM-MSAP	9	55	245	7
19	35/M	25	N	N	2	Sarcoma resection	Knee	GM-MSAP	63	80	150	9
20	29/M	25	N	N	2	Sarcoma resection	Knee	GM-MSAP	180	140	150	15
21	42/M	25	N	N	2	Sarcoma resection	Knee	GM-MSAP	140	130	130	18

Abbreviations: ASA, American Society of Anesthesiologists; BMI, body mass index; F, female; GM-LSAP, chimeric gastrocnemius musculocutaneous–lateral sural artery perforator flap; GM-MSAP, chimeric gastrocnemius musculocutaneous–medial sural artery perforator flap; M, male; N, no; OM, osteosynthesis material; Pt, patient; TKA, total knee arthroplasty; Y, yes.

### Orthopaedic Patients

The average age at surgery was 64 years old (range: 27–88).

One- and two-stage procedures were equally distributed in the group (8 patients each). Defect size following the debridement was 52 cm ^2^ on average (range: 9–140), whereas the mean size of the skin paddle was 73 cm ^2^ (range: 20–129). A chimeric musculocutaneous GM-MSAP was used in 15 patients, whereas in 1 patient an LSAP was used. The donor site was closed primarily in 7 patients, whereas in the remaining 9 a skin graft was necessary. The mean follow-up was 18 months.

Flap reraise/readvancement was necessary in 11 patients due either to a recurrent infection of the material (5 cases) or a planned two-stage procedure (6 cases; Table 2 ).

**Table 2:** Flaps-related characteristics

Pt no.	Ortho strategy/oncology	Type of framework	Donor site closure	Flap complications	Persistent infection	Need for reraise	Successful reraise	Further plastic surgery
1	I Stage	TKA	STSG	None	N	N	N/A	N
2	I Stage	OM	Direct	None	Y	Y	Y	N
3	I Stage	TKA	STSG	None	N	Y	Y	N
4	II Stage	OM	STSG	None	N	N	N/A	N
5	I Stage	TKA	Direct	None	N	N	N/A	N
6	II Stage	Spacer	Direct	None	N	Y	Y	N
7	I Stage	Spacer	STSG	Wound dehiscence	N	Y	Y	N
8	Oncology	N/A	STSG	Partial flap necrosis	N/A	N/A	N/A	Lateral GM
9	II Stage	Spacer	STSG	None	N	Y	Y	N
10	II Stage	TKA	Direct	None	N	Y	Y	N
11	II Stage	Spacer	Direct	None	N	Y	Y	N
12	I Stage	TKA	STSG	None	Y	Y	Y	N
13	Oncology	N/A	Direct	None	N/A	N/A	N/A	N
14	II Stage	Spacer	STSG	None	N	Y	Y	N
15	I Stage	TKA	Direct	None	Y	Y	Y	N
16	I Stage	TKA	STSG	None	Y	N	N/A	N
17	I Stage	OM	STSG	Superficial skin necrosis	N	N	N/A	N
18	II Stage	Spacer	Direct	None	Y	Y	Y	N
19	Oncology	N/A	Direct	None	N/A	N/A	N/A	N
20	Oncology	N/A	STSG	None	N/A	N/A	N/A	N
21	Oncology	N/A	STSG	None	N/A	N/A	N/A	N

Abbreviations: GM, gastrocnemius muscle; N/A, not applicable; OM, osteosynthesis material; N, no; STSG, split thickness skin graft; TKA, total knee arthroplasty; Y, yes.

Flap reraise associated to further implant exchange or extensive debridement/washout was successful in all cases, without need for any further flap procedure. Limb salvage was achieved in all cases.

Among complications, we recorded 1 case of flap wound dehiscence and 1 case of distal flap necrosis which were managed successfully with conservative treatment without need of further procedures. One case of donor site infection was recorded following skin graft and was addressed with a superficial debridement and a new STSG.

### Oncological Patients

The flap coverage was performed following the confirmation of the pathological margins of resection. Defects ranged from 50 to 200 cm ^2^ (average: 126) with a skin paddle average of 122 cm ^2^ (range: 80–180). A GM-MSAP was used in 4 out of 5 cases and a GM-LSAP was used in the remaining. The donor site was closed primarily in 2 cases, whereas in 3 patients a skin graft was used. On average, the follow-up was of 18 months.

Among complications, a distal necrosis of the MSAP skin paddle in patient nr. 8 led to reexposure of bone element around the knee and a muscular lateral gastrocnemius was necessary to ensure stable coverage ( Table 3 ).

**Table 3:** Comparison between orthopaedic and oncological patients

	Orthopaedic Pt	Oncological Pt
Number of patients	16	5
Mean age (y)	64	34
MSAP/LSAP	15/1	4/1
Mean defect size (cm ^2^ )	52	126
Mean skin paddle size (cm ^2^ )	73	122
Mean operative time (min)	198	228
Mean follow-up (mo)	18	18
Major complications	0	1
Minor complications	3	0

Abbreviations: MSAP/LSAP, medial sural artery perforator/lateral sural artery perforator; Pt, patient.

## Discussion

The lack of soft tissue availability and skin redundancy at the knee level represent a well-known issue and makes local options relatively limited in terms of size and flap component. Despite evolution in prosthetic surgery and microbiological innovations, persistent infection and multiple reoperations are often necessary in case of implant infection, arming the viability of the local soft tissue around the knee. 15

Muscle flap coverage is often required in such situation either as a prophylactic treatment or during revision surgery. Despite being a workhorse flap in such context, GM is sometimes insufficient to reach superolateral and supra patellar defects, being sometimes particularly narrow or tendinous in its distal part, making coverage less stable. Moreover, the need to skin grafting makes postoperative immobilization and healing longer. 16 17 18 Even if closer to the knee apex, distally based muscle flaps are either insufficient (sartorius) or retain unacceptable donor site morbidity (e.g., vastus lateralis). 18 Fasciocutaneous flaps alternatives from the knee area have been proposed, such as medial or lateral superior genicular artery perforator flaps or saphenous flaps. 17 However, these flaps are generally reduced in size and can reach anterior tibial tuberosity with difficulty. Reverse-flow anterolateral thigh flaps has been proposed as effective solution for superior and superolateral defects, but the risk of venous congestion should be considered as it may potentially impair the reconstruction, especially if framework material or TKA is present. 2

The MSAP flap, 19 despite being an elegant solution for superficial knee resurfacing, has some relevant disadvantages in arthroplasty procedures, such as difficult harvesting with perforator intramuscular dissection, longer operative time, 16 and, mostly, the lack of a reliable muscle component to fill dead space and guarantee adequate vascular perfusion around the knee implant.

Although musculocutaneous gastrocnemius has been described long time ago, 7 literature shows how it did have limited success, probably due to its bulkiness and inset difficulties when harvesting the whole skin over the calf area.

However, composite principles and perforator/propeller flaps recently opened new tailoring possibilities in complex knee reconstructions. Indeed, composite flaps can incorporate multiple tissue types (e.g., muscle and skin), which are potentially connected only by branching or perforating vessels. A chimeric GM-MSAP flap can therefore serve for the double aim of filling a deep defect, while at the same time addressing skin shortage with the fasciocutaneous component. 20 21 22

As mentioned, the skin paddle component allows to reach cranial and superolateral defects which are generally out of the reach of a simple GM flap. This potential is further enhanced in case of more distal perforators entering the skin and eccentric placement of the skin paddle in relation to the perforator. This chimeric solution is particularly fit in those defects involving the tibial tuberosity (covered by the muscle) and the patella, covered by the skin paddle rotated in a propeller fashion. 23

In our experience, this chimeric flap has revealed to be particularly powerful when addressing implant-associated defects. In the first step of two-stage TKA revisions, the presence of the skin paddle enables a wider coverage, with increased quantity of antibiotic cement to be delivered. In the second step, the skin paddle allows for more elasticity over the knee and an easier access to the ortho team, minimizing the risks of suture tension and breakdown.

It should be considered that TKA revisions are extremely complex as they often imply multiple reinterventions on the same patient, with persistent infectious risk, despite the surgical strategy chosen (one vs. two stages). The presence of a solid skin paddle and therefore acquired skin availability and laxity enables the surgeon multiple flap reraises when needed.

It has been reported that up to 0.5% of patients with TKA will finally return to theater after early wound healing complications. 1 Among these, more than 50% incur into a deep wound infection after TKA. This translates into a percentage of over 5% of required supplementary major additional surgery in the long term. 23

When dealing with relevant oncological defects around the knee, both muscle and skin components could be used to resurface the whole area without the need of a free flap. Despite expanding the GM coverage size possibilities, this chimeric modification does not replace the role of free flaps, as sovra-dimentioned skin paddles may incur in partial necrosis, as happened in patient nr. 8.

Mayoly et al described the use of the conventional GM flap in a series of 16 soft tissue knee reconstruction, leaving the cutaneous portion attached to the underlying muscle. 17 Their results are similar to those experienced in this study; however, our surgical technique involves the individualization and dissection of the most distal suitable perforator to allow us to propeller the skin paddle over the muscle and gain as much arc of rotation as possible and increase the surface of the flap.

Importantly, it needs to be noticed that in roughly 10% of cases, MSA perforators are not eligible for a safe perforator–propeller skin flap harvest. 24 In our series, when basing a relevant skin paddle on a small-sized perforator we incurred in a superficial skin paddle necrosis, which may result in secondary flap procedures if defects to be covered are particularly extended. We believe that the surgical procedure should always start with the exploration of the available perforator through an anterior approach: in case of lack of suitable perforators, a standard GM-only flap can be easily performed as backup solution without adding further scars.

Importantly, besides perforator size, perforator's position should be carefully evaluated. Basing the skin paddle MSAP on perforators too proximal to the gastrocnemius pivot point will reduce the potential flap length or require a distal extension of the skin paddle with the potential risk of vascular insufficiency. 25

According to our experience the microsurgical skill level required for the chimeric MSAP flap harvest is relatively low. The perforator intramuscular dissection is usually limited and the plane between the subcutaneous tissue and the deep fascia (where the perforators are encountered) is easily dissected bluntly, minimizing the risk of vascular damage.

On the other hand, the learning curve is steeper when it comes to flap inset at the recipient site, especially in complicated knees with multiple previous surgical accesses and scars. Flap design need to be accurately planned on perforator position, specifically when a skin bridge is present between the flap tibial harvesting incision and the defect. When designing the flap, the distance between the flap pedicle pivot point (in the popliteal fossa) and the proximal part of the skin paddle will generally correspond to the width of the cutaneous bridge. This will reduce flap bulk under the bridge and will allow to place the skin component only where needed.

We acknowledge the retrospective nature of this study, the relatively low number of cases and the lack of a comparative cohort of patients (e.g., GM-only flap).

Nevertheless, this study represents to our knowledge the largest series in literature using a chimeric musculocutaneous GM-MSAP/LSAP propeller–perforator flap, with a relevant number of patients presenting knee defects associated to osteosynthesis material and TKA. This allowed evaluating GM-MSAP flaps performances during a long-term follow up, not only in terms of simple quality of coverage, but more importantly in terms of facility of flap reraise in case of repeated surgeries over time.

This specific flap tool translates in a critical advantage in orthoplastic surgery of the knee and when dealing with repeatedly infected TKA or frameworks requiring coverage.

## References

[1] Galat D D McGovern S C Larson D R Harrington J R Hanssen A D Clarke H D Surgical treatment of early wound complications following primary total knee arthroplasty J Bone Joint Surg Am 2009 91 01 48 54 19122078 10.2106/JBJS.G.01371

[2] Sapino G Zaugg P Cherix S ALT flap with vascularized fascia lata for one-stage functional patellar tendon reconstruction J Plast Reconstr Aesthet Surg 2019 72 03 467 476 30579912 10.1016/j.bjps.2018.11.002

[3] Rao J Tawar R Dawar R Gastrocnemius myocutaneous flap: a versatile option to cover the defect of upper and middle third leg World J Plast Surg 2018 7 03 314 318 30560070 10.29252/wjps.7.3.314 PMC6290304

[4] Gkiatas I Korompilia M Kostas-Agnantis I Tsirigkakis S E Stavraki M Korompilias A Gastrocnemius pedicled muscle flap for knee and upper tibia soft tissue reconstruction. A useful tool for the orthopaedic surgeon Injury 2021 52 12 3679 3684 33892927 10.1016/j.injury.2021.04.009

[5] Panse N Bhadgale R Karanjkar A Phulwer R Sahasrabudhe P Ramteke C The reach of the gastrocnemius musculocutaneous flap: how high is high? World J Plast Surg 2018 7 03 319 325 30560071 10.29252/wjps.7.3.319 PMC6290316

[6] Daigeler A Drücke D Tatar K The pedicled gastrocnemius muscle flap: a review of 218 cases Plast Reconstr Surg 2009 123 01 250 257 19116559 10.1097/PRS.0b013e3181904e2e

[7] McCraw J B Fishman J H Sharzer L A The versatile gastrocnemius myocutaneous flap Plast Reconstr Surg 1978 62 01 15 23 351647 10.1097/00006534-197807000-00002

[8] Hallock G G The medial sural artery perforator flap: a historical trek from ignominious to “workhorse” Arch Plast Surg 2022 49 02 240 252 35832674 10.1055/s-0042-1744425 PMC9045491

[9] Hallock G G The medial sural (medial gastrocnemius) perforator local flap Ann Plast Surg 2004 53 05 501 505 15502470 10.1097/01.sap.0000116338.59679.12

[10] Peng P Dong Z Wei J Modified lateral gastrocnemius myocutaneous flap with extended anterior and/or inferior boundary Sci Rep 2022 12 01 1031 35058537 10.1038/s41598-022-05093-2 PMC8776792

[11] Tande A J Patel R Prosthetic joint infection Clin Microbiol Rev 2014 27 02 302 345 24696437 10.1128/CMR.00111-13 PMC3993098

[12] Müller S LC Morgenstern M Kuehl R Soft-tissue reconstruction in lower-leg fracture-related infections: an orthoplastic outcome and risk factor analysis Injury 2021 52 11 3489 3497 34304885 10.1016/j.injury.2021.07.022

[13] di Summa P G Sapino G Cherubino M Reconstruction of complex soft tissue defects including tendons with anterolateral thigh flap extended to fascia lata: long term recovery and functional outcomes Microsurgery 2019 39 05 405 415 30672005 10.1002/micr.30431

[14] Saint-Cyr M Wong C Schaverien M Mojallal A Rohrich R J The perforasome theory: vascular anatomy and clinical implications Plast Reconstr Surg 2009 124 05 1529 1544 20009839 10.1097/PRS.0b013e3181b98a6c

[15] Izakovicova P Borens O Trampuz A Periprosthetic joint infection: current concepts and outlook EFORT Open Rev 2019 4 07 482 494 31423332 10.1302/2058-5241.4.180092 PMC6667982

[16] Cho Y J Lee J H Chung D W Pedicled chimeric gastrocnemius-medial sural artery adipofascial flap for reconstruction of anterolateral defects of the knee Microsurgery 2017 37 03 206 211 26095849 10.1002/micr.22436

[17] Mayoly A Mattei J C Moullot P Gastrocnemius myocutaneous flaps for knee joint coverage Ann Plast Surg 2018 81 02 208 214 29762447 10.1097/SAP.0000000000001451

[18] Swartz W M Ramasastry S S McGill J R Noonan J D Distally based vastus lateralis muscle flap for coverage of wounds about the knee Plast Reconstr Surg 1987 80 02 255 265 3602175 10.1097/00006534-198708000-00016

[19] Cavadas P C Sanz-Giménez-Rico J R Gutierrez-de la Cámara A Navarro-Monzonís A Soler-Nomdedeu S Martínez-Soriano F The medial sural artery perforator free flap Plast Reconstr Surg 2001 108 06 1609 1615, discussion 1616–1617 11711936 10.1097/00006534-200111000-00027

[20] Hallock G G Chimeric gastrocnemius muscle and sural artery perforator local flap Ann Plast Surg 2008 61 03 306 309 18724133 10.1097/SAP.0b013e31815b2792

[21] Hallock G G The chimeric propeller flap Semin Plast Surg 2020 34 03 207 209 33041692 10.1055/s-0040-1714290 PMC7542215

[22] Cavadas P C Teran-Saavedra P P Combined latissimus dorsi-thoracodorsal artery perforator free flap: the “razor flap” J Reconstr Microsurg 2002 18 01 29 31 11917953 10.1055/s-2002-19706

[23] Innocenti M Cardin-Langlois E Menichini G Baldrighi C Gastrocnaemius-propeller extended miocutanous flap: a new chimaeric flap for soft tissue reconstruction of the knee J Plast Reconstr Aesthet Surg 2014 67 02 244 251 24211051 10.1016/j.bjps.2013.10.011

[24] Dusseldorp J R Pham Q J Ngo Q Gianoutsos M Moradi P Vascular anatomy of the medial sural artery perforator flap: a new classification system of intra-muscular branching patterns J Plast Reconstr Aesthet Surg 2014 67 09 1267 1275 24957803 10.1016/j.bjps.2014.05.016

[25] Teo T C The propeller flap concept Clin Plast Surg 2010 37 04 615 626, vi 20816517 10.1016/j.cps.2010.06.003

